# Novel Injectable Hydrogel Formulations and Gas Chromatography Analysis of the Residual Crosslinker in Formulations Intended for Pharmaceutical and Cosmetic Applications

**DOI:** 10.3390/gels10040280

**Published:** 2024-04-21

**Authors:** Fatimah Rashid, Paul Carter, Stephen Childs

**Affiliations:** School of Pharmacy and Pharmaceutics, Faculty of Health Sciences and Wellbeing, University of Sunderland, Sunderland SR1 3SD, UK; paul.carter@sunderland.ac.uk

**Keywords:** injectable hydrogels, filler, hyaluronic acid, cosmetic, aesthetic, rheology, SEM, GC analysis, residual crosslinker

## Abstract

Novel hyaluronic acid (HA) crosslinked with pentaerythritol tetra-acrylate (PT) injectable hydrogels was invented. These injectable hydrogel/dermal filler formulations were synthesised using HA and the acrylate PT as a crosslinker under basic pH conditions using thermal crosslinking methods (oven heating), which provides a simple, safe, and eco-friendly method for crosslinking in 4 h under 45 °C. Fourier-transform infrared spectroscopy (FTIR) and scanning electron microscopy (SEM) analyses were conducted to represent the difference between the formulations in terms of peak formation and pore size, respectively. The crosslinking was partial as is considered to be typical for dermal injectable fillers. The rheological properties of these formulations showed that these novel dermal injectables are highly promising, and the newly developed fillers could be used with better results for dermal anti-wrinkle corrections, shaping, and volumising reasons. Furthermore, crosslinker (PT) residual analysis was carried out to state the formulations that are valid and acceptable for intradermal usage. The results from the GC method validation revealed it was a suitable method for this study. The GC analysis of all five injectable hydrogel/filler formulations demonstrated the formulations HA-PT 1, 2, 3 and 4 were formulated using (0.05–0.1)% *w*/*w* PT containing residual PT monomers within the safe limits that were determined to be below (0.008% *w*/*w*). This work has shown the development of a novel injectable hydrogel/filler formulation for pharmaceutical and cosmetic applications can be prepared in a more sustainable and simple way using pentaerythritol tetra-acrylate as a crosslinker agent, which holds great promise for the industry’s future advancement.

## 1. Introduction

The use of injectable fillers has gained widespread popularity in soft tissue augmentation and reshaping the face, curves and contours and giving dimension to achieve the proper aesthetic goals to fulfil the concept of beautifying [1]. These dermal injectable fillers are used for skin youth restoration and in anti-wrinkle products; these gels are implanted inside the derm and restore volume [2,3,4]. Most areas that are beautified are on the face, such as lip reshaping, giving volume, removing lines, etc. [4]. Moreover, the degree of correction varies from fine to moderate to severe [2]. Fine corrections mostly are superficial, including the lip submucosa, tear trough, and periorbital regions, while moderate corrections of the mid dermis include such things as midface volume and forehead lines. Lastly, severe modifications can go to the deep dermis such as the cheeks, chin, and lateral brow, medial brow, and jawline [2,5,6].

Injectable hydrogels may also be utilised in other applications rather than beautifying, such as wound healing, biomedical applications, and the delivering of drugs to targeted sites [7,8,9]. Injectable HA hydrogels are commonly used in various biomedical applications due to their unique properties. These hydrogels serve as effective carriers for therapeutic agents, offering benefits like minimal invasiveness, adaptability to irregular sites, and biocompatibility [10]. They can be tailored to control the release of therapeutic agents, enhancing the treatment efficacy in diseases, cancers, and tissue regeneration [11]. The sol–gel transition of these hydrogels allows for the precise tuning of the network morphology and properties to regulate drug delivery, making them promising biomaterials for scaffolds and carriers in the biomedical field [10,12]. The versatility of HA hydrogels extends to applications in skin injury repair, angiogenesis, and targeted drug delivery systems [13]. In general, the design and utilisation of injectable HA injectable hydrogels represent a significant advancement in biomedical research, offering a promising avenue for enhanced therapeutic outcomes across various medical applications.

The recent fillers that are the gold standard and unique for soft tissue filling and beautification are HA-based injectable hydrogel filler formulations [14,15]. As known, HA-based cosmetic formulations, such as gels, autologous fatty gels, dermal/intra-dermal filler injections, creams, hydrogels, patches, lotions, and serums, provide greatly noticeable space-filling, anti-wrinkle, skin anti-aging, anti-nasolabial-folding, and major face-rejuvenating properties [16,17,18]. However, achieving optimum results from the potential cosmetic application of HA for a long duration is still challenging due to some of the undesirable chemical properties.

**Figure 1:**
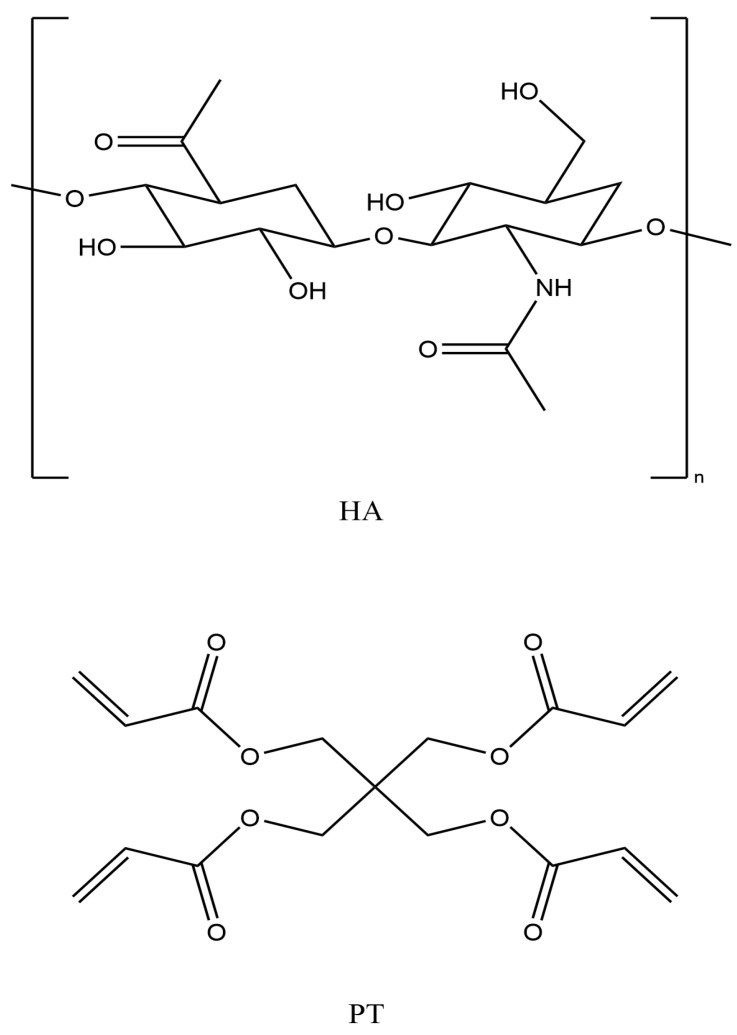
Hyaluronic acid (HA) and pentaerythritol tetra-acrylate (PT) chemical structures (drawn with ChemDraw, version 16).

HA is biodegradable and exhibits suboptimal efficacy due to its inability to form physically linked hydrogels over variable pH conditions [16]. Furthermore, HA is a natural, biodegradable polymer with a short half-life of around 12 h as it undergoes rapid degradation by the hyaluronidase enzymes present in body tissues [19,20]. Therefore, it needs to be chemically crosslinked with a safer crosslinker to overcome these limitations and provide better results in some cosmetic applications. This will reduce the enzymatic degradation rates even if crosslinking is partial compared to those of linear polymers due to the presence of covalent bridges and intermolecular bonds/forces between the polymer chains and the chemical crosslinker [16,21]. HA has three different functional groups available (hydroxyl (-OH), carboxylic (-COOH) and amide (-NHCOCH3) for crosslinking via different mechanisms or reactions, such as an ether linkage (R-O-R), ester bond (R-COO-R) and carbodiimide, respectively [9] (see Figure 1). Previously, many crosslinkers, such as 1-ethyl-3-(3-dimethylaminopropyl) carbodiimide (EDC), glutaraldehyde (GTA), poly (ethylene glycol) diglycidyl ether (PEGDE), ethylene glycol diglycidyl ether (EGDE) and divinyl sulfonate (DVS), have been used to crosslink HA [19]. However, to ensure the biocompatibility and safety of crosslinked HA filler formulations, the effective proportion of a crosslinker in a formulation should be as low as possible.

The new HA crosslinked fillers in this commercial field and in the market are crosslinked with BDDE, DVS (divinyl sulfone), biscarbodiimde and methacrylate [1].

PT (pentaerythritol tetra-acrylate) is a tetra-functional acrylate monomer that is mostly used as a crosslinker in polymerisation reactions [16,22] (Figure 1). It is a viscous and colourless liquid, with a density of 1.19 g/mL, and it is immiscible with water [23,24]. PT has also been used to crosslink HA hydrogel films [22,23,25]. It is widely used as a solvent, a colouring agent and a fragrance in pharmaceutical and cosmetic applications [16]. The earlier studies have used PT as a crosslinker for polyethylene oxide (PEO) via UV radiation and in an alginate hydrogel formulation [22,23,25]. Recently, PT was used to crosslink HA hydrogel via exposure to high temperatures (80 °C) in an oven to produce a completely crosslinked film [16]. Applying a temperature of 45 °C can enable partial crosslinking, whilst leaving the formulation in an aqueous form rather than converting to a film form. Accordingly, this study included a 45 °C crosslinking reaction temperature. However, this crosslinking reaction may result in the synthesised gel formulation containing low moieties of aromatic impurities of the residual crosslinker [26]; therefore, it is important to prevent the presence of such multifunctional acrylate monomers in injectable hydrogel/filler formulations. Unreacted acrylate monomers can cause skin irritation and other serious side effects, such as allergies, inflammation, dermatitis and skin sensitisation [22]. Hence, most dermal fillers are partially crosslinked.

Gas chromatography (GC) and high-performance liquid chromatography (HPLC) are the predominant methods employed for assessing residual monomers in quality control analysis due to their selectivity and compatibility. Additionally, other spectroscopic techniques, such as nuclear magnetic resonance (NMR) spectroscopy, have been utilised for quantifying the residual acrylate monomers. However, GC analysis was used in this study for residual crosslinker detection in the injectable hydrogel/filler formulations due to its accuracy, convenience, versatility and sensitivity for the precise quantification of chemicals [27,28].

The aims of this study are as follows: (i) New injectable hydrogel/filler formulations crosslinked with PT for the first time and different formulations synthesised with different HA polymer percentages and variable crosslinker PT amounts are invented.

Each formulation could be used for various pharmaceutical applications, such as therapeutic delivery, biomedical applications and tissue engineering.

For cosmetic applications non-surgical facial rejuvenation provides natural-looking results with minimal discomfort and a short recovery time. These formulations will be useful to apply for different corrections within applications, including volume restoration, smoothing out wrinkles, lip augmentation and enhancing the facial contours.

(ii) The residual PT crosslinker that is left uncrosslinked in the injectable hydrogel/filler formulations are detected and quantified by using GC analysis.

## 2. Results and Discussion

### 2.1. Crosslinking Mechanism

**Figure 2:**
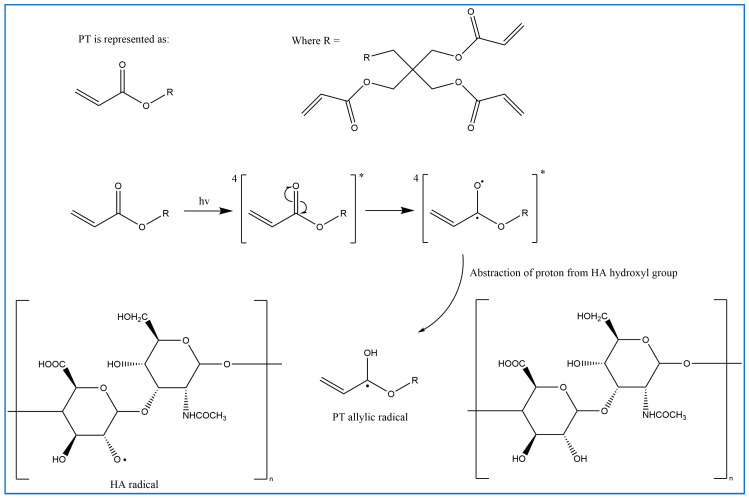
Proposed radical polymerisation mechanism induced via UV and heat between HA and PT (drawn using ChemDraw, version 16).

**Figure 3:**
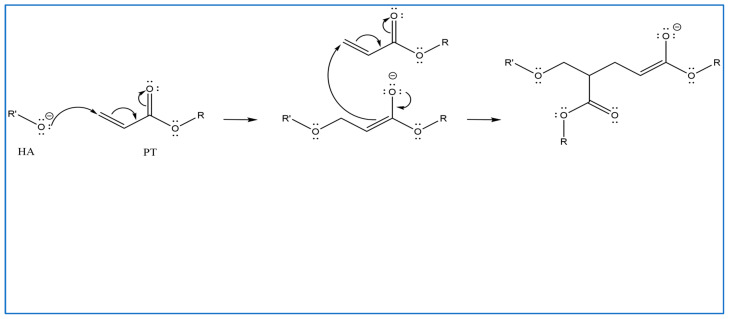
Proposed Michael addition reaction by the activation of the -OH of PT and nucleophiles generation under alkaline conditions; they generate electrons or protons in this Michael reaction (drawn using ChemDraw, version 16).

**Figure 4:**
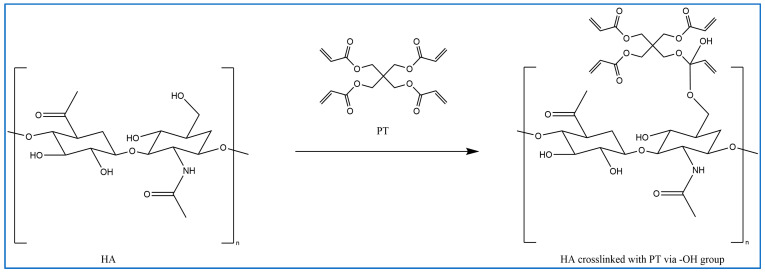
Proposed methacrylation process: crosslinking between HA and PT polymers via the -OH group of the HA and carbonyl carbon of the PT (drawn using ChemDraw, version 16).

In previous studies, PT has been used as a crosslinker of polyethylene oxide (PEO) hydrogel films synthesised using UV radiation [29]. Other studies suggest that when PT absorbs energy from UV photons, its acrylate group generates reactive-free radicals, which initiates the crosslinking of repeated units of the polymer with PT radicals via recombination [29]. However, it is difficult to induce the free radicals on PT without adding a photoinitiator. Therefore, in this study, the reaction mechanism was described in most of the reactions where possible. Firstly, for UV- or heat-induced PT radicals, which abstract a hydrogen atom from HA to generate HA radicals for repeated HA units that recombine with PT radicals to form crosslinks despite the absence of an initiator in the procedure, PT possibly forms free radicals at a higher temperature [30,31,32] (Figure 2). The crosslinking of PT and HA radicals could occur via the hydroxyl (-OH) group of HA and the carboxylic (-COOH) group of the PT. However, with this suggestion, the pH turns alkaline without any input, which is improbable. The second suggested mechanism is that when PT is activated by -OH as nucleophiles generated under alkaline conditions, they generate electrons or protons via a Michael reaction (Figure 3). The other possible reaction suggested is the acrylation of HA under an alkaline condition similar to the methacrylation process, which successfully occurs by activating a carbon–carbon double bond [32] (Figure 4). Since a reaction between HA and PT has not been reported before, we explained the most acceptable reaction mechanisms. However, the injectable hydrogel/filler formulation was suggested to be partially crosslinked. Lastly, to achieve our aims, we intend to perform a future follow-up and continue to conduct more comprehensive in-depth research on the mechanism of this new crosslinking reaction.

### 2.2. Rheology Study of the Injectable Hydrogel/Filler Formulations

One of the main studied areas of injectable hydrogel/filler is investigating the viscoelastic properties of dermal injectable hydrogels and fillers [9]. The HA-PT formulations were analysed with an oscillatory rheometer, which is usually characterised by the linear viscoelastic region, storage (elastic) modulus G′ and the loss (viscous) modulus G″ (*n* = 3), mostly determining the flowability and applied stress [33]. In addition, these parameters are valuable details that could apply to all injectable hydrogel/filler formulations [20].

The linear viscoelastic region (LVR) is a measure of the shear elastic moduli of the sample. However, the LVR was determined for all the HA-PT injectable hydrogel/filler formulation samples via the calculation of the elastic moduli G′ and viscose moduli G″ over the amplitude sweep of the shear stress.

**Figure 5:**
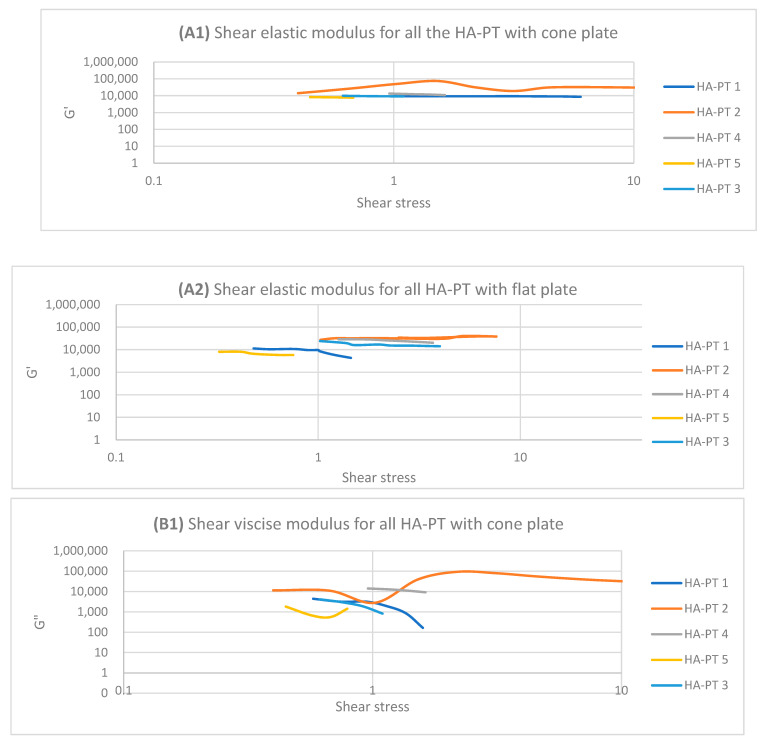

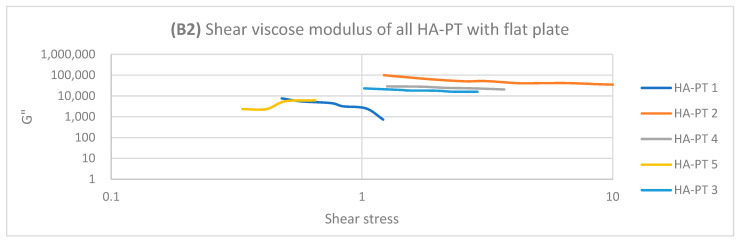
Logarithmic plot of the amplitude sweep for all HA-PT 1, 2, 3, 4 and 5 formulations with cone and flat plates. (**A1**) Shear elastic modulus (G′) plot vs. shear stress with cone plate. (**A2**) Shear elastic modulus (G′) plot vs. shear stress with flat plate. (**B1**) Shear viscose modulus (G″) plot vs. shear stress with cone plate. (**B2**) Shear viscose modulus (G″) plot vs. shear stress with flat plate.

The HA-PT 2 injectable hydrogel/filler formulation appeared to have a shorter LVR compared to HA-PT 1 on both the cone and parallel flat plates due to HA-PT 1 having a lower concentration of HA and PT, offering fewer viscoelasticity properties (Figure 5), while G′ and G″ showed increasing in viscosity upon increasing the frequency with the frequency sweep. In general HA-PT 2 showed better viscoelastic properties than HA-PT 1, with G′ 33,587.49 ± (859.68) and G″ 48,154.24 ± (754.55) on the parallel flat plate, which are also in agreement with those on the cone plate, with 34,715.63 ± (1321.29) for G′ and 40,004.39 ± (262.82) for G″, as shown in Table 1 and Table 2.

**Figure 6:**
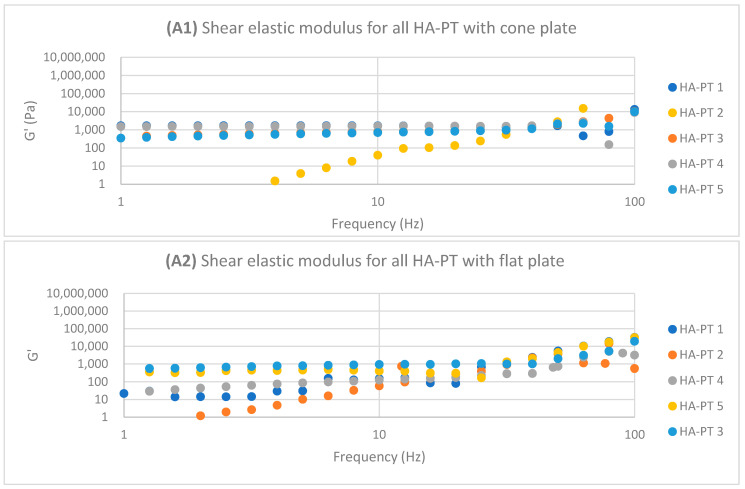

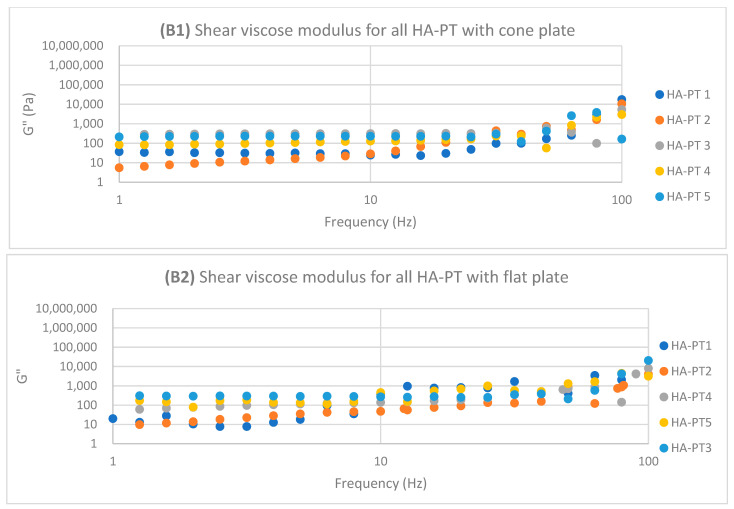
Logarithmic plot of the frequency sweep for all HA-PT 1, 2, 3, 4 and 5 formulations with cone and flat plates. (**A1**) Shear elastic modulus (G′) plot vs. frequency with cone plate. (**A2**) Shear elastic modulus (G′) plot vs. the frequency with flat plate. (**B1**) Shear viscose modulus (G″) plot vs. frequency with cone plate. (**B2**) Shear viscose modulus (G″) plot vs. frequency with flat plate.

Regarding HA-PT 4 and HA-PT 5, they both exhibited more viscoelastic behaviour, with both the cone and parallel flat plates appearing more structured than the other formulations; this could be due to the higher percentage of crosslinker in the injectable hydrogel/filler formulation. Thus, they both showed a shorter LVR with the cone plate. However, they showed less dependency on the frequency during the frequency sweep toward G′ and G″. The HA-PT 4 formulation’s G′ and G″ were similar on both the plates, while those of HA-PT 5 appeared more elastic, with G′ being higher than G″ (6591.36 ± (232.28); 4268.30 ± (58.26), respectively), with the flat plate being similar to the cone plate (G′ 7678.64 ± (313.98); G″ 1180.44 ± (129.02)) (see Figure 6). 

The HA-PT 3 formulation represented higher G′ and G″ moduli on the parallel flat smooth plate in both the amplitude and frequency sweeps than on the cone plate (Figure 5 and Figure 6). Also, the cone plate measurement for HA-PT 3 after the amplitude sweep reflects a drop in elasticity to near viscosity after 10.

Overall, the injectable hydrogel/filler formulation rheological measurement results appeared to have viscose moduli G″ that are higher or closer to the elasticity modulus G′, suggesting that these hydrogel formulations were in gel form and typical for injectable usage.

The shear viscosity complex component η* (Pa s) is a frequency-dependent value that measures the elastic and viscous properties of materials. Mostly solid materials have a higher η* value that indicates the reluctance to flow. However, the formulations’ η* appeared to decrease as the frequency increased, which indicates the injectable hydrogel/filler formulation samples’ shear thinning [34,35].

In this study, both the parallel flat plate and cone plate were used to compare the results; specially, these novel formulations were studied for the first time, and they both provide results that are different from each other. Both the plates are applicable for these formulations, but the cone plate provides a constant shear rate, which compared to the parallel plate [36], undergo less stress.

### 2.3. Injectable Hydrogel/Filler Formulation Characterisation

**Figure 7:**
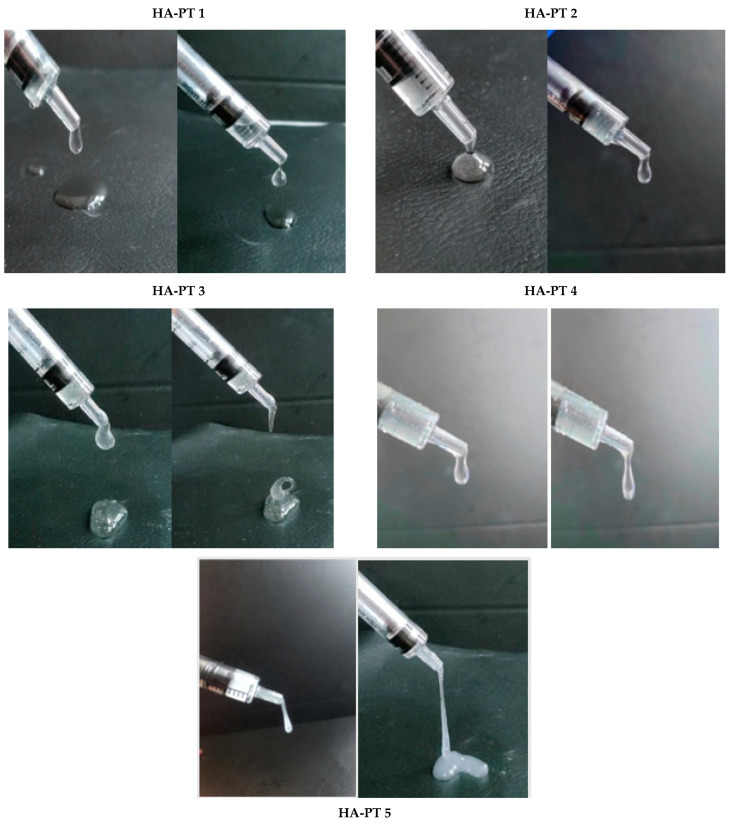
Images of each (HA-PT) filler formulation demonstrating the viscosity and elasticity of the formulations while the syringe is being compressed, and a closer view showing the viscoelastic properties of the formulations.

The physical, visual appearance and flowability in the syringe, the viscosity and the thickness of the HA-PT injectable hydrogel/filler formulations are shown below in Figure 7. It also provides visual evidence of the formulation coming out of the syringe. Wongprasert et al. [2] represented the viscosity of the injectable hydrogel/filler schematically; our study provides actual images of the formulations with varying viscosities, which are promising for a wide range of applications. HA-PT 1 appeared to have the lowest viscosity comparing to all the other formulations, while the HA-PT 3 had the highest viscosity. HA-PT 4 and 5 appeared to be more elastic due to the higher % of crosslinker, and this was also confirmed with a rheological study.

### 2.4. Formulations and Swelling Behaviour of the Injectable Hydrogel/Filler Formulations

Five formulations named HA-PT 1, 2, 3, 4 and 5 were formulated with different concentrations of HA and PT, as summarised in (Table 3).

**Figure 8:**
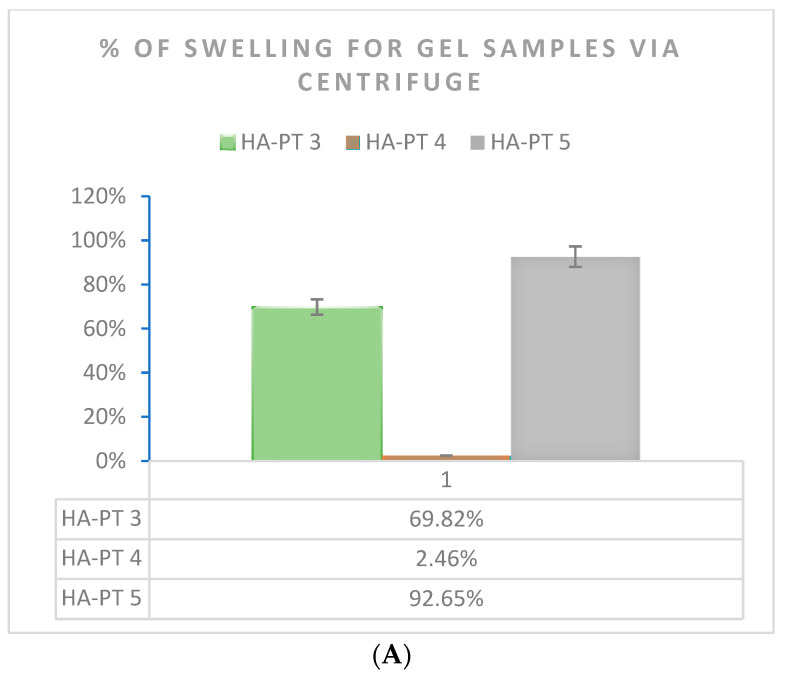

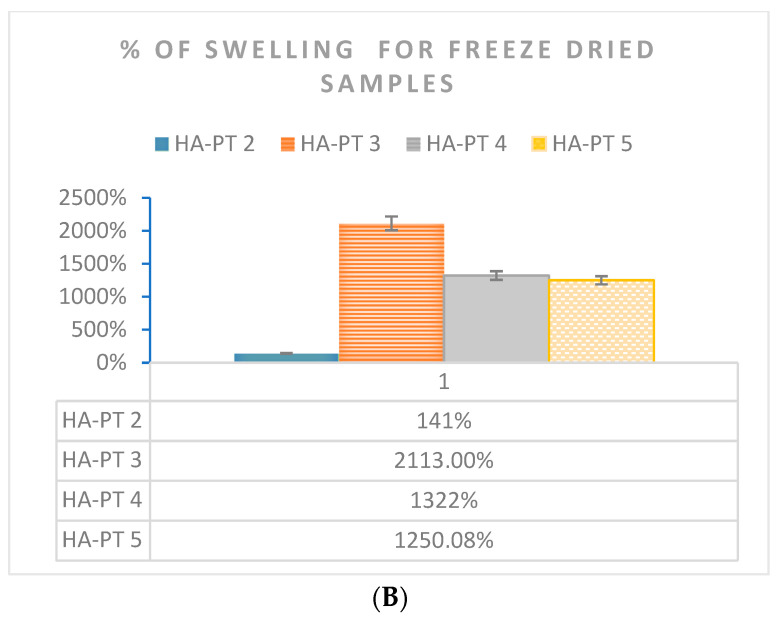
Swelling % of HA-PT gel formulation samples *n* = 3 (**A**) determined by centrifuging the gel formulations samples and (**B**) determined by a swelling test for freeze-dried samples.

Due to the importance of the swelling capacity of the injectable hydrogel/filler formulations used as injectable formulations [33], in this study, swelling was studied using two methods (*n* = 3) (Figure 8). The formulations exhibited remarkable swelling due to the HA polymer having hydrophilic groups in its chemical structure, which allowed the gel-formed hydrogel formulation to absorb water and be distinctively hydrated [9]. As expected, the swelling percentage of HA-PT 5 (20 mg HA + 25% PT) was the highest % at around 92.65% compared to that of HA-PT 4 (20 mg HA + 5% PT). This is because a higher amount of (PT) crosslinker could enable the formulation to have more water uptake inside the structure and expand more as a hydrogel [16]. However, HA-PT 3 represented 69.82% of the swelling as a more viscous, condensed gel structure. However, for HA-PT 1 and 2, it was difficult to determine the % of swelling, which could be due to the difficulty to isolate the swelled hydrogel as HA-PT 1 had a lower concentration of HA and a low % of crosslinker.

Regarding the swelling study via freeze drying prior to the swelling of the injectable hydrogel/filler samples, it was found that HA-PT 3 had (2113%) a higher % of swelling than all the other HA-PT samples; this is explained due to the higher concentration of HA, which means it was able to absorb more water in the structure, while the low crosslinker ratio imparted a less-elastic structure [33]. Moreover, the HA-PT 2 sample showed 140.80% swelling, which was the lowest among the HA-PT 3, 4 and 5 samples. However, the main reason for the higher % of swelling after freeze drying in comparison to the gel centrifuge method is that freeze drying the samples dries them, so they do not have any moisture in their dry structure, and during the swelling process, they take more water to their structure to convert to a gel form. Therefore, the concentration of HA polymer has a large impact on the swelling ratio, as shown in Figure 8. HA-PT 1 was difficult to measure the swelling of it because having a low HA concentration impeded the isolation of the swelled supernatant. Many researchers have chosen to study swelling using either method; therefore, we selected both methods for the swelling study to easily compare data and provide a wider understand of both the methods [33,37].

### 2.5. FTIR Study

**Figure 9:**
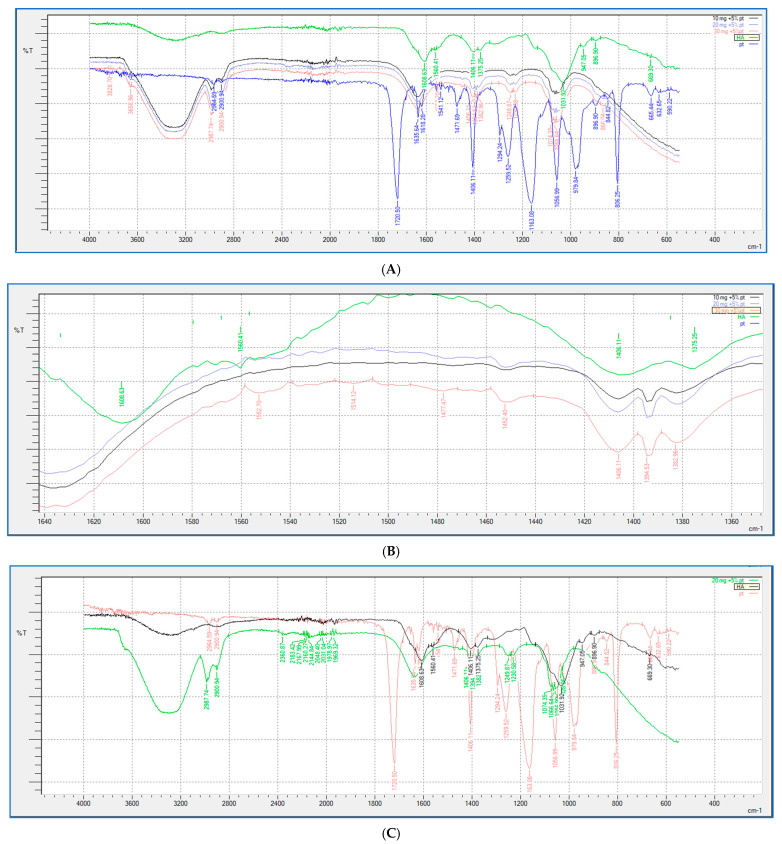

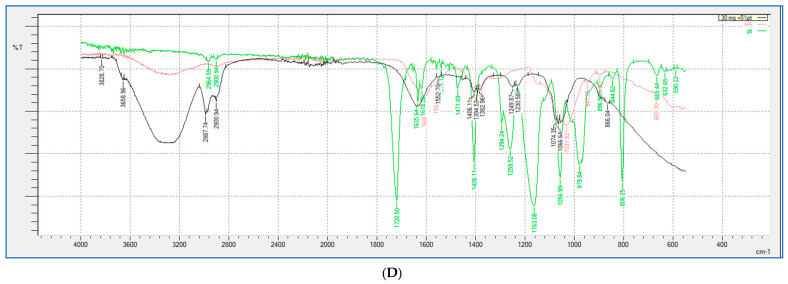
Showing (**A**) FTIR spectra of pure HA, HA-PT 1, HA-PT 2, HA-PT 3 and pure PT. (**B**) Enlarged FTIR spectra of pure HA, HA-PT 1, HA-PT 2, HA-PT 3 and pure PT. (**C**) FTIR spectra of pure HA, HA-PT 2 and pure PT. (**D**) FTIR spectra of pure HA, HA-PT 3 and pure PT.

**Figure 10:**
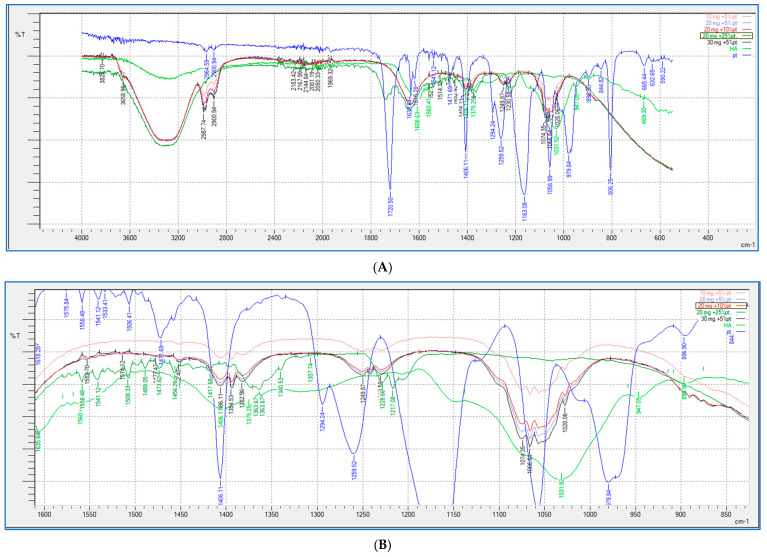
Showing (**A**) FTIR spectra of pure HA, HA-PT 1, HA-PT 2, HA-PT 3, HA-PT 4, HA-PT 5 and pure PT. (**B**) Enlarged FTIR spectra of pure HA, HA-PT 1, HA-PT 2, HA-PT 3, HA-PT 4, HA-PT 5 and pure PT.

FTIR analysis (shown in Figure 9 and Figure 10) was performed for all the HA-PT injectable hydrogel/filler formulations, pure HA and pure crosslinker (PT), to explore the molecular interactions inside the formulation polymer matrix [38]. Figure 10 illustrates the FTIR spectra of all the HA-PT injectable hydrogel/filler formulations, pure HA and pure PT, which show HA polymer crosslinking with PT via possibly an ester bond (C-O-C) between the hydroxyl group of the HA and the carbonyl carbon of the crosslinker PT present around 1050–1100 cm^−1^. Moreover, the stretching peak around 1100–1300 cm^−1^ referring to (C-O-C) is the new bond (crosslink) between HA and PT in the injectable hydrogel/filler formulations. By looking more closely at the stretched peak around 1050–1100 cm^−1^, in each injectable hydrogel/filler formulation sample, which is represented in the enlarged (Figure 10B), it appears that HA-PT 1 and 2 stretched the least, while HA-PT 4 and 5 had a more-stretched peak due to the larger concentration of crosslinker in these injectable hydrogel/filler formulations. However, the HA-PT 3 formulation’s peak (Figure 9D) was more stretched; this is because the higher concentration of HA in the formulation with more functional groups in the HA allowed it to uptake of all the PT added in the formulation without leaving any PT monomer uncrosslinked. This is similar for HA-PT 2 (Figure 9C). On the other hand, the peaks around 2900–3300 cm^−1^ shown for in all the HA-PT injectable hydrogel/filler formulations stretched, suggesting a polymer hydroxyl vibration region [39].

Generally, all the HA-PT injectable hydrogel/filler formulations exhibited partial crosslinking in comparison to the hydrogel films that fully crosslinked [16], which denotes that these injectable hydrogel/filler formulations are suitable to be dermal injectable fillers, and each formulation could be used for specific application that vary from fine to moderate to deep corrections during anti-wrinkle applications [2].

### 2.6. SEM

**Figure 11:**
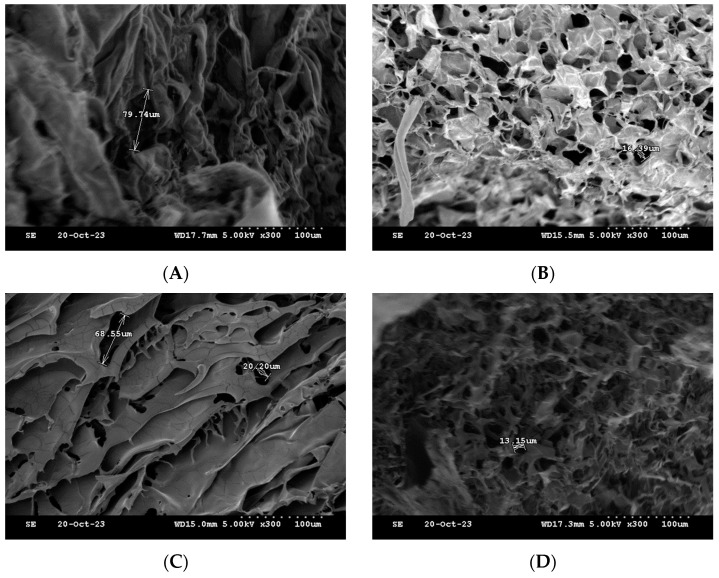

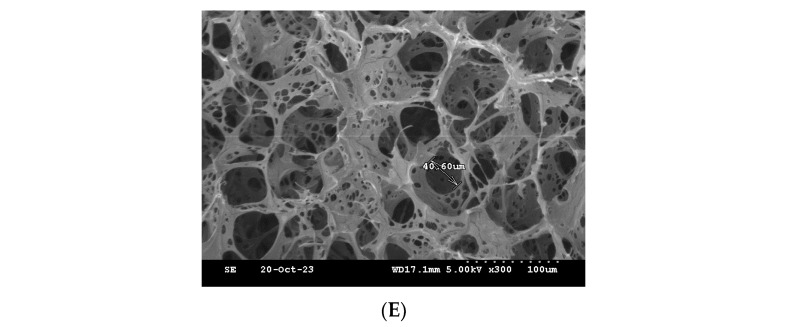
Cross-section SEM images of the freeze-dried filler formulations. (**A**) HA-PT 1. (**B**) HA-PT 2. (**C**) HA-PT 3. (**D**) HA-PT 4. (**E**) HA-PT 5. The scale bar represents ×300 (100 µm).

One of the commonly used techniques for assessing injectable hydrogel/filler formulations is SEM. This technique allows for studying the porous microstructure, distribution and agglomeration of the polymeric matrices [33] (Figure 11). The microstructure of the formulations was observed, and the pores sizes were also determined for the freeze-dried formulations. The formulations HA-PT 1, 2 and 3 appeared to have a different microstructure than each other; specially, these three formulations (HA-PT 1, 2 and 3) had the same concentration of PT with different HA concentrations. HA-PT 3 had a structure more like folds, which referred to a dense HA polymer in Figure 11C. In addition, the increase in PT % in the formulation caused a decrease in pore size [33]; this was obvious for the formulations HA-PT 2 with the pore size 16.39 µm and HA-PT 4 with the pore size 13.15 µm. The SEM results are in agreement with the results of the swelling study since the two formulations mentioned above had the lowest % of swelling, whereas the % of swelling of HA-PT 2 was 141 (±20.99)% with the freeze drying method, and HA-PT 4 had the lowest % of swelling of about 2.46 (±0.60)% with the centrifuge process. However, HA-PT 5 had a larger pore size (40.60) µm despite having a higher crosslinker % in the formulation, which suggests that the formulation has a high residual PT content, and which also appeared to have the highest swelling % via centrifuging of 92.65 (±3.55) %. Overall, the pore size cannot be solely relied upon as it is not exact; it is approximate.

### 2.7. GC Analysis of the Residual Crosslinker on the Novel Injectable Hydrogel/Filler Formulations

#### 2.7.1. Overview of the Crosslinker

According to GHS, PT has been classified as a skin sensitiser [40]. An earlier in vivo study determined that the patches that were made from PETA (pentaerythritol tri-acrylate) caused serious skin sensitising in human and Guinea pig skin [41]. According to the previous studies on PT, the maximum acceptance of PETA was 0.01% (*m*/*v*), Notably, it was reported that PT is less-skin-sensitising than PETA [22,42].

An injectable hydrogel/filler formulation mostly contains pure HA. If a crosslinker is used in these formulations, they are mostly partially crosslinked, in which case, a small amount of the crosslinker should be added and must have no/a low amount of any residual content.

**Figure 12:**
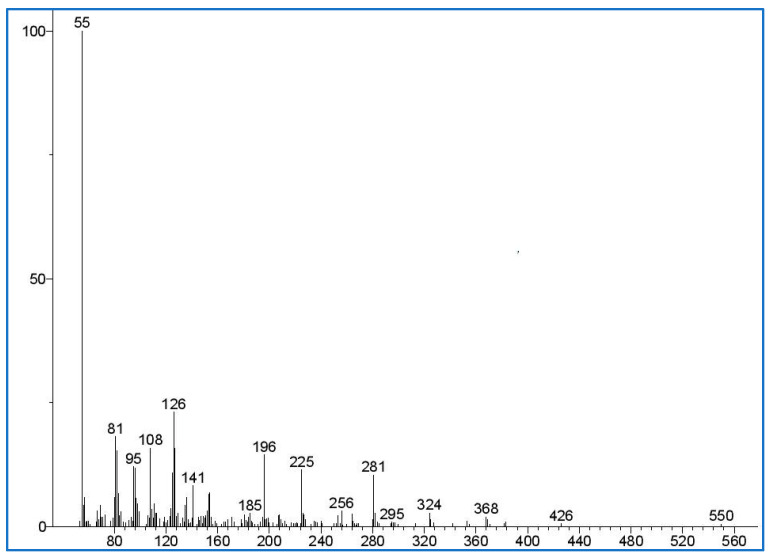
This represents the mass spectra of pentaerythritol tetra-acrylate (PT) detected in the sample extraction solution. The *y*-axis of the mass spectra is the signal intensity (in abundance or counts arbitrary units); the *x*-axis (*m*/*z*) is the mass-to-charge ratio of the detected signal.

The previous studies have documented the maximum acceptable residual PT concentration based on the polymer used to produce a hydrogel. Accordingly, in this study, the maximum acceptable residual PT and crosslinker concentration after recalculating using the density of the used polymer (HA; 1.80 g/mL) was found to be 0.008% *w*/*w* [22]. This amount is valid for hydrogel formulations after taking in account the polymer density. The molecular mass of the PT ion was identified by using GC-MS spectra (Figure 12)**,** and it matches the mass spectra in the literature [43]. HexA was selected as a suitable internal standard solution as it shares a similar chemical structure with PT, and its retention time does not interfere with the PT peaks.

#### 2.7.2. Gas Chromatography Method

**Figure 13:**
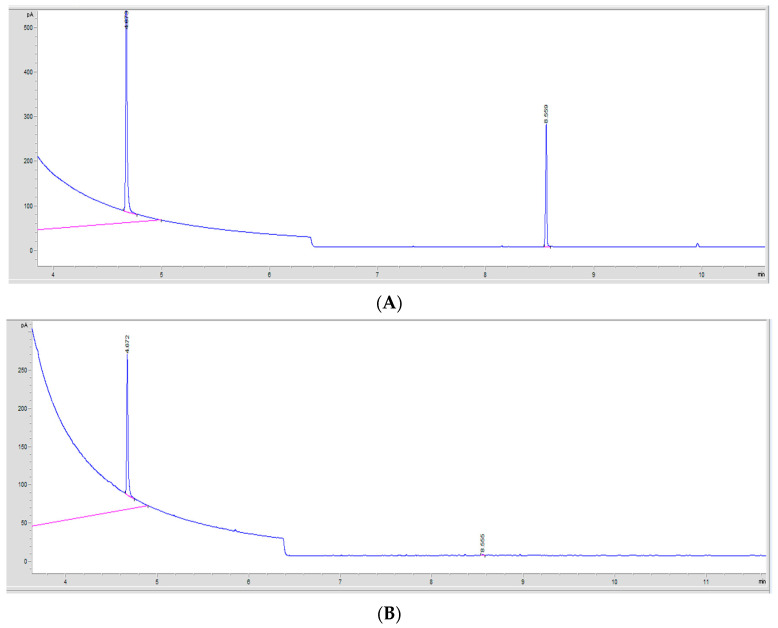
The GC chromatograms of the detected PT monomers. (**A**) A GC chromatogram showing the PT peak detected in a standard solution. The PT monomer peak at 8.55 min retention time (RT). The IS internal solution (HexA) peak was at 4.67 min retention time (RT). (**B**) A GC chromatogram showing the PT peak in a sample extraction solution of residual PT in the formulations. The crosslinker (PT) monomer peak was at 8.55 min retention time (RT), while the IS internal solution (HexA) peak was at 4.66 min retention time (RT).

To detect and quantify the residual PT that was left uncrosslinked in the injectable hydrogel/filler formulations, GC analysis was used. HA-PT formulation sampling was carried out using two methods: directly using the formulation and using the freeze-dried samples prior to extraction. The sample extraction method with the GC method was adapted from [22]. The obtained chromatograms are presented in Figure 13. The validity of the GC method was assessed using the method validation criteria explained further in the sections below.

Chloroform was chosen as a suitable volatile solvent to dissolve the PT, and the internal standard used was hexylacrylate (HexA) [44]. However, the PT and HexA (internal solution) peaks were at 8.55 min ± 0.006 and 4.67 min ± 0.001 retention time (RT), respectively [29]. Although, there were a few peak fractions corresponding to PT on the gas chromatograms, only the most-abundant peak at 8.55–8.52 min was used for analysis during this study.

##### Response Linearity

**Figure 14:**
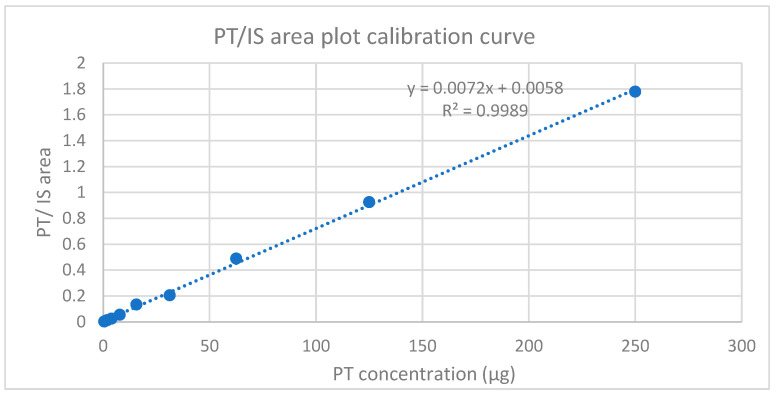
Calibration curve plot for PT standard solutions with HexA (internal solution).

The good linear relationship shown in Figure 14 between the peak area ratio of PT to HexA (IS) vs. the corresponding concentration was determined from ten standards ranging from 0.0166 to 0.000032% *w*/*w*, as detailed in the methodology, Section 4.9.3 Standard Solutions and the Calibration Curve. However, below the figure, the calibration curve obtained from the mean of three injections of each extracted injectable hydrogel/filler sample solution with a regression coefficient (R^2^ > 0.998) is shown, which was used to determine the LOD and LOQ [45]. In addition, the obtained calibration curve concentration range covered a wider range of limit of the residual PT.

##### Precision

In this study, the relative standard deviation (RSD) of the PT peak area ratio of repeated measurements of the samples was assessed to determine the precision of the used GC method for residual PT analysis. Six injections of the system suitability were measured on the same day as intra-day precision and on three different days as inter-day precision. The RSD% for intra-day precision was 3.54%, and for inter-day precision, it was 2.95% (Table 4). The obtained RSD values were within the accepted limit of 15% [46], thus proving that this method is precise and reproducible for such analysis.

##### Accuracy

For the accuracy of this study, the GC method was used for the detection of extracted residual PT in the injectable hydrogel/filler formulations, calculated among three equal spiked samples (*n* = 3) that were prepared with three different PT concentrations. The amount of PT in the blank samples was 0.00014% *w*/*w*, and this was used as a reference.

The percentage of the accurate recovery was determined using Equation (1) [29].

(1) $$\;\%\;of\;accurate\;recovery=\frac{C_{recoverd}}{C_{spiked}+C_{blank}}\times 100$$

where the *C_recovered_* is the PT concentration calculated in the spiked samples (% *w*/*w*), *C_spiked_* refers to the PT concentration that added to the spiked samples (0.0166, 0.00833 and 0.00103)% *w*/*w*, while the *C_blank_* refers to the PT concentration in the blank sample, which was 0.00014% *w*/*w*.

According to the literature [22,29], both the % recovery and the relative standard deviation (RSD%) should be within the limit of 80–120% recovery and (±15)% RSD, respectively [22,29]. However, in the study, the data appear to have a correspondence between the obtained results and the method’s suitability for quantifying the PT’s residual concentration in the HA-PT formulations, and Table 5 shows that the mean % of accuracy recovery was (91.72)% on average and the relative standard deviation (RSD%) was 0.77%, which proves the method’s reliability and efficiency.

##### Sensitivity

Using GC for analysis, the limit of detection (LOD) and limit of quantitation (LOQ) are important values of accuracy and indicate the method’s validity [46]. Both the limit of detection (LOD) and limit of quantitation (LOQ) were evaluated based on the signal-to-noise ratio (S/N) in the PT standards [47]. Furthermore, the accepted LOD from the S/N value should be over three (>three) [48]. While the LOQ obtained from the sharp peak confirms the lowest concentration, which resolves >10% of the baseline for a sensitivity study [49].

In this study, to ensure the method’s sensitivity, the LOD and LOQ values were determined, and Table 5 represents the PT concentrations corresponding to the LOD value (0.000032% *w*/*w*) and the LOQ value (0.00013 % *w*/*w*). Thus, the LOD value was 3.13, and LOQ value was 19.93, demonstrating that the method is highly sensitive (Table 6).

##### Robustness

One of the essential parts of a high-quality assurance system in GC analysis studies is a robustness study [49,50]. This will ensure the method is reliable. Many parameters were included in this robustness study, such as the oven temperature (±5), changes in the detector temperature (±5) and the flow rate (±10%). Three different PT concentrations samples were measured in triplicate, as tabulated in Table 7.

The RSD% results that obtained were confirmed that the peak area ratio for all the extracted samples with three PT concentrations was reproducible, with %RSD = 2.294. This method’s suitability and robustness depend on the relative standard deviation (RSD) value for the cumulative sample concentration, which must not exceed 15% [22]. Based upon this criterion, the GC method was deemed to be suitably robust.

## 3. Conclusions

In this study, novel injectable hydrogel/filler formulations (HA-PT 1, 2, 3, 4 and 5) were synthesised, which consist of different HA concentrations and vary in the degree of crosslinking with different concentrations of crosslinker. These novel injectable hydrogels/fillers were synthesised from HA using PT as a crosslinker for the first time. The injectable hydrogel/filler formulations vary in firmness, which allow them to be used for different types of dermal correction. HA-PT 1 and 2 could be used as fine and soft injectable hydrogels/fillers as superficial lip submucosa and to reshape the lips, remove lateral canthal lines, etc., while HA-PT 3 could be used for moderate-to-severe augmentations, such as lateral brow, cheekbone, jawline and cheek augmentations, etc. HA-PT 4 and 5 with higher PT amounts are more useful for more-severe and deep dermis corrections due to their increased firmness.

Regarding other pharmaceutical and biomedical applications, the HA-PT 1, 2 and 4 formulations are more suitable for tissue engineering because of the thinner structure. In addition, these formulations are modified and crosslinked HA, which addresses the limitation of native HA and allows them to be more suitable for the biomedical field. Therefore, these HA injectable hydrogels formulations are promising in delivering therapeutic drugs, cells or biologics for tissue repair, making them a valuable tool in regenerative medicine applications. Their biocompatibility and ability to encapsulate cells, which contribute to their effectiveness in promoting tissue regeneration, are our research focus in a future follow-up.

Fillers (injectable dermal hydrogels) are used as a volumiser and shaper for soft tissues. Mostly, these fillers are either pure HA without a crosslinker or have HA and are partially crosslinked. Moreover, only a few crosslinkers, such as BDDE, DVS and methacrylate, have been reported to be used successfully as crosslinkers, and pentaerythritol tetra-acrylate has not been used as a crosslinker in injectable hydrogel/filler formulations. Tetra acrylates are strong skin irritants and sensitisers; therefore, it is important to quantify the levels of residual acrylate monomers in the formulation, especially when a formulation will be released under the skin by injection. The level of residual crosslinker must be within the accepted range to be safe. The development of these new injectable hydrogel/filler formulations also required residual PT analysis. The results from GC analysis were valid and showed a high level of precision for residual acrylate analysis. All of the HA-PT formulations contained significantly less than the acceptable amount of residuals, except HA-PT 5, which had a high residual amount of the crosslinker, most likely due to the high concentration (0.5%) *w*/*w* of PT added in this formulation (Table 8). The amount of residual PT monomer detected and extracted from the freeze-dried samples was similar to the samples that were directly used for extraction, which suggests that detection was valid for the only uncrosslinked residual PT without considering any PT monomers that were crosslinked. This result suggests that freeze drying the samples does not negatively affect stability in terms of PT release, and therefore it might be feasible to manufacture these injectable hydrogel/filler formulations in a freeze-dried form for subsequent re-hydration, allowing for better shelf-life stability. This study concluded that the injectable hydrogel/filler formulations HA-PT 1, 2, 3 and 4, which were formulated using 0.05–0.1% *w*/*w* PT, contained residual PT monomers below 0.008% *w*/*w*; therefore, they are likely to be safe for dermatological injectable hydrogel/filler use. Overall, we have successfully developed novel injectable hydrogel/filler formulations that are made from natural polymer HA and crosslinked with PT, and they were crosslinked using a simple, easy, safe and sustainable method. Furthermore, they have promise for a variety of applications in both the pharmaceutical and cosmetic fields. Our aim for future work is to investigate the hyaluronidase enzyme hydrolysis profile, assessing the ability of hyaluronidase to degrade the filler in various clinical scenarios.

## 4. Materials and Methods

### 4.1. Materials and Chemicals

HA (HA) sodium salt with a high molecular weight (1800–2200 KDa) was supplied by Infinity Ingredients (Binfield, UK), while pentaerythritol tetra-acrylate (PT) was purchased from Insight Biotechnology Limited (Middlesex, UK). Hexylacrylate (Hex) was purchased from Sigma-Aldrich (Gillingham, UK). These materials were used as received unless otherwise described. The other chemicals and reagents included NaOH (1.0 M), which was used for pH adjustment. Deionised distilled water was available in the laboratory and was used as a solvent for HA polymer gel making and as a pore-swelling agent for the HA-PT crosslinked fillers. Chloroform was used as the extraction solvent for residual crosslinker analysis.

### 4.2. Synthesis of the HA-PT Injectable Hydrogel/Filler Formulations

HA-PT injectable hydrogel/filler formulations were prepared with different concentrations of HA and PT, as summarised in Table 3. The formulations were prepared by dissolving HA in deionised distilled water. The mixtures were stirred with an IKA stirrer (IKA^®^ Werke GmbH & Co. KG, Staufen, Germany) from 3 h to 24 h according to the HA concentration to obtain homogeneous HA formulations. This was followed by increasing the pH of the formulations to 11 using NaOH and adjusting the pH by using a pH meter from Hanna Instruments, a wireless pH tester, and then adding different amounts of PT, where the mixture was subsequently stirred slowly for 24 h to obtain completely homogenised HA-PT formulations.

### 4.3. Crosslinking Experiment

The HA-PT hydrogel films were subjected to oven-assisted thermal crosslinking. The formulations were put in the 45 °C oven (Binder GmbH Berg ster, 14 D-78532 Tuttlingen) for 4 h for the crosslinking reaction (thermally assisted crosslinking) of the HA-PT injectable hydrogel/filler formulations. The obtained formulations were used for many analyses.

### 4.4. Rheological Properties Study of the Injectable Hydrogel/Filler Formulations

The oscillatory rheological properties of all the HA_PT injectable hydrogel/filler formulations were measured with a Malvern Kinexus rotational rheometer (Malvern Instruments Ltd., Malvern, UK) equipped with two different adaptors. First, a parallel flat smooth adaptor (20 mm) was used to heat a stainless-steel parallel plate to 25 °C. The injectable hydrogel/filler samples were fitted between the upper parallel plate and a stationary surface. Second, a cone plate adaptor CP4/40 PL65 and a stainless-steel cone plate were used. The gap size for the injectable hydrogel/filler samples was set to 0.1 on a parallel flat plate and with cone plate. The elastic modulus (G′) and viscous modulus (G″) were determined as a function of shear strain (amplitude sweep) and as a function of frequency (frequency sweep). The linear viscoelastic region (LVR) was estimated by performing an amplitude sweep test at incremental shear strains (from 1 to 100 Pa) and a fixed frequency of 1 Hz, while the frequency sweep of injectable hydrogel/filler samples was performed within the LVR under a specific shear strain (γ) for each HA_PT injectable hydrogel/filler formulation sample and at decreasing oscillating frequencies from 100 to 1 rad/s Hz.

### 4.5. Freeze Drying

The samples from each formulation were freeze-dried. Prior to freeze drying, the samples were directly frozen in a −80 °C freezer, and subsequently freeze-dried in a VIRTIS BENCHTOP PRO device from SP SCIENTIFIC, Stone Ridge, NY, USA, for 24 h to thoroughly remove the water. The freeze-dried samples were used in the swelling test, for SEM and for the extraction of chloroform for GC analysis.

### 4.6. Swelling Study

Two methods were used to study the swelling of the injectable hydrogel/filler formulations.

#### 4.6.1. Centrifuging of the Swelled Gel Samples

The formulations’ swelling behaviours were studied by centrifuging the samples. An accurately weighed (*Md*) quantity of injectable hydrogel/filler gel samples from each formulation was swelled in distilled water in centrifuge tubes for 24 h at room temperature. The swelled mixture was centrifuged for 30 min at 14,500 rpm, and the supernatant was removed and reweighed (*Ms*). The percentage of swelling (%) was calculated using Equation (2) [2].

(2) $$\%\;of\;swelling=\frac{Ms-Md}{Md}\times 100$$

where *Md* is the initial gel weight and *Ms* is the weight of the swollen gel at equilibrium.

#### 4.6.2. Swelling Study to Freeze-Dried Samples

The injectable hydrogel/filler formulations’ swelling behaviours were studied by swelling the freeze-dried sample. An accurately weighed (*Md*) quantity of freeze-dried gel samples from each formulation was swelled in distilled water for 24 h at room temperature. Later, the excess water was removed from the swelled samples, and the swelled samples were reweighed (*Ms*). The percentage swelling (%) was calculated using Equation (2) [2].

### 4.7. Fourier-Transform Infrared Spectroscopy (FT-IR)

FT-IR analysis was carried out at room temperature using a Shimadzu IR Affinity-1S Fourier-Transform Infrared Spectrometer (Shimadzu UK Ltd., Milton Keynes, UK) for all the HA_PT injectable hydrogel/filler formulation batches to evaluate the crosslinking degree of HA-PT formulations. FT-IR analysis was also performed for pure HA and pure PT for comparison. The spectral range was 4000–550 cm^−1^, with a resolution of two wave numbers (cm^−1^).

### 4.8. SEM

The morphology and pore sizes of the injectable hydrogel samples were evaluated using a scanning electron microscope (Hitachi, Tokyo, Japan) operated in high vacuum mode at an accelerating voltage of 5 kV. Prior to freeze drying, the samples were frozen in a −80 freezer, and then they were freeze-dried in an ALPHA 2–4/LSC device (Martin Christ Gefriertrocknungsanlagen GmbH, Osterode am Harz, Germany) under a vacuum of 0.1 Pa at −70 °C for 24 h to thoroughly remove water. However, HA is a hygroscopic compound when it is in a dry form; therefore, the vials were filled with nitrogen gas to keep the samples away from moisture. Furthermore, the freeze-dried formulations samples were placed into liquid nitrogen for a one minute, and then a razor blade was used to cut them in a way to allow for the internal structures to be exposed and show the pores more clearly. Then, they were applied onto the sticky surface of the sample holder. All the samples were sputter-coated with gold and palladium using an Agar spurrer coater (AGAR-Scientific, Ltd., Essex, UK) for 60 s before observation.

### 4.9. Residual Crosslinker (PT) Analysis in the Injectable Hydrogel/Filler Formulations

#### 4.9.1. Gas Chromatography Analysis

The PT residual analysis of the extracted HA-PT injectable hydrogel/filler formulation solutions was carried out using an Agilent Technologies 7890A Gas Chromatograph (Agilent Technologies, Santa Clara, CA, USA). Chromatographic separation was carried out on a fused-silica capillary column (30 mm × 0.32 mm × 0.25 mm) coated with 5% phenyl-methyl-polysiloxane. The injector was used in splitless mode, and its temperature was maintained at 300 °C during separation, while the column temperature ranged from 50 °C (hold time = 1 min) to 280 °C (hold time = 4 min) at a rate of 30 °C/min. The carrier gas (helium) flow rate was 2 mL/min.

In this study, the samples are represented in *w*/*w* % by converting all the concentrations from *w*/*v* to *w*/*w* % using Equation (3) [22,51].

(3) $$\%\;of\;solute\;(\%\;w/w)=\frac{amount\;of\;solute\left(g\right)}{amount\;of\;solution\left(g\right)}\times 100$$

where the amount of solute (PT), the crosslinker, is the extracted PT amount from the samples in (g), while the amount of the solution refers to the solvent used for extraction, taking in account the density of the solvent, which was chloroform (1.5 g/mL).

#### 4.9.2. Internal Standard Solution

An internal standard solution is widely used in GC analysis, which allow for analyte recovery during sample preparation and instrumental analysis [30]. In this study, hexylacrylate (HexA) was used as an internal standard. Precisely 25 mg of HexA was weighed and diluted with 50 mL of the solvent (chloroform), and then 1 mL of the resulting solution was diluted with 10 mL chloroform to a final concentration of 50 μg/mL. The peak area of 50 μg/mL of the HexA internal standard fell around the middle point of the obtained calibration curve. Importantly, this HexA internal standard solution was then included in all the prepared standards for calibration curve generation and also in the sample solutions.

#### 4.9.3. Standard Solutions and the Calibration Curve

Firstly, a stock solution of PT was prepared by dissolving 25 mg in 50 mL of chloroform to obtain 500 μg/mL concentration. Then, ten standard solutions with PT concentrations ranging from 250, 125, 62.5, 31.25, 15.625, 7.81, 3.90, 1.95, 0.97 to 0.48 μg/mL were prepared. Each standard concentration had specific % concentrations (0.0166%, 0.00833%, 0.00416%, 0.0020%, 0.0010%, 0.00052%, 0.00026%, 0.00013%, 0.000065% and 0.000032% (*w*/*w*)) and were prepared using the stock solution. They were poured into 2 mL disposable vials to ensure homogenous mixing, and then mixed in an overhead shaker. Lastly, 400 μL from each prepared standard was placed into a GC vial, along with 100 μL of (HexA) internal standard solution. All the standard solutions were measured in triplicate. Calibration curves were drawn by plotting the obtained results using the peak area of PT and the peak area of HexA.

#### 4.9.4. Sample Preparation for Extraction

Sampling and GC analysis were performed using different methods.

1. Directly using the jelly injectable hydrogel/filler formulations.
2. Using freeze-dried samples for PT extraction.

From each HA-PT gel formulation and freeze-dried HA-PT sample, an adequate amount approximately weighing 0.0100 g was individually transferred into glass vials, and 1 mL chloroform was extracted for 24 h at room temperature. The vials were tightly closed and left in the shaking water bath (80 rpm, temperature 25 °C) for the 24 h extraction. Later, the extracted solution was filtered to remove the gel base formulation or freeze-dried sample, and pure extracted chloroform was used for GC analysis. A total of 100 μL of the internal solution was added to 400 μL extracted solution in GC vials, and they were injected directly to the GC in triplicate.

Notably, all the samples were extracted directly without washing and dialysis in order to find the actual amount of uncrosslinked PT directly in the formulation that was left as a residual.

### 4.10. Statistical Analysis

One-way analysis of variance (ANOVA) was used to compare rheological parameters mean shear stress, G′, G″, and η* between the formulations. Differences within and between treatments were significant at an acceptable *p*-value of <0.05. Relative standard deviation (RSD)% and mean ± SD was applied in GC analysis results.

## Figures and Tables

**Table 1:** Rheological parameters for the formulations with flat plate. *p*-value for all the data < 0.05 reflects a significant difference between the variants. *n* = 3.

Formulations	Elastic Modulus G′ (Pa)	Viscous Modulus G″ (Pa)	Mean Shear Stress (Pa) in LVR Amplitude Sweep	Shear Viscosity Complex Component η* (Pa s)
**HA-PT 1**	8789.88 (±50.62)	4561.09 (±231.43)	0.8968 (±0.03)	523.48 (±18.35)
**HA-PT 2**	33,587.49 (±859.68)	48,154.24 (±754.55)	5.0562 (±0.76)	558.44 (±32.22)
**HA-PT 3**	16,577.38 (±1006.46)	17,493.79 (±989.99)	1.9533 (±0.23)	3380.83 (±222.48)
**HA-PT 4**	24,547.31 (±984.54)	24,905.16 (±1500.26)	2.2613 (±0.21)	712.41 (±7.65)
**HA-PT 5**	6591.36 (±232.28)	4268.307 (±58.26)	0.5042 (±0.01)	2807.13 (±70.37)

**Table 2:** Rheological parameters for formulations with cone plate. *p*-value for all data < 0.05 reflects a significant difference between variants. *n* = 3.

Formulations	Elastic Modulus G′ (Pa)	Viscous Modulus G″ (Pa)	Mean Shear Stress (Pa) in LVR Amplitude Sweep	Shear Viscosity Complex Component η* (Pa s)
**HA-PT 1**	9298.62 (±147.41)	1270.76 (±146.77)	1.1411 (±0.06)	2.3993 (±0.37)
**HA-PT 2**	34,715.63 (±1321.29)	40,004.39 (±262.82)	4.4946 (±0.17)	2.5922 (±0.18)
**HA-PT 3**	9313.16 (±8.88)	2258.15 (±186.05)	0.7983 (±0.04)	24.5479 (±1.31)
**HA-PT 4**	13,714.86 (±8082.77)	11,458.35 (±1053.20)	1.5095 (±0.41)	50.7569 (±22.01)
**HA-PT 5**	7678.64 (±313.98)	1180.44 (±129.02)	0.6255 (±0.03)	22.9670 (±1.93)

**Table 3:** Summary of the formulations with HA concentrations and PT %.

Filler Formulations Names	HA Concentration Added (mg/mL)	The % *w*/*w* of PT in the Formulation	%*w*/*w* of the PT in HA
**HA-PT 1**	10 mg	0.05%	5%
**HA-PT 2**	20 mg	0.05%	5%
**HA-PT 3**	30 mg	0.05%	5%
**HA-PT 4**	20 mg	0.1%	10%
**HA-PT 5**	20 mg	0.5%	25%

**Table 4:** RSD% represented in precision results from the intra-day and inter-day analyses of selected PT concentrations.

Selected PT Concentrations (% *w*/*w*)	Peak Area Ratio % (Mean ± SD) Intra-Day Precision	RSD%	Peak Area Ratio % (Mean ± SD) Inter-Day Precision	RSD%
**0.000032**	0.28 (±0.005)	2.00	0.282 (±0.001)	0.47
**0.001000**	12.04 (±0.238)	1.98	13.095 (±0.875)	6.68
**0.016600**	106.29 (±7.071)	6.66	110.304 (±1.870)	1.70
**Mean**		(3.54)		(2.95)

**Table 5:** The accuracy data of the PT sample extraction at 3 different PT concentrations.

Selected PT Concentrations Added % *w*/*w* (Spike)	Blank Sample Concentration % *w*/*w*	Recovered Concentration % *w*/*w*	% Recovery (Accuracy)	%RSD
**0.0166**	0.00014	0.01553	92.75 (± 0.51)	0.56
**0.00833**	0.00014	0.0078	92.05 (± 1.52)	1.65
**0.00103**	0.00014	0.001	90.35 (± 0.08)	0.09
	**Mean**		**91.72**	**0.77**

**Table 6:** Represent the limit of detection (LOD) and limit of quantitation (LOQ) of PT (*n* = 3).

Sensitivity Parameters	PT Concentration % *w*/*w*	Signal of Noise N/S (±SD)	RSD%
**LOD**	0.000032	3.13 (± 0.05)	1.84%
**LOQ**	0.000130	19.93 (± 0.11)	0.57%

**Table 7:** All this study’s robustness results for PT concentration with different parameters. For each parameter, *n* = 3.

Different Parameters	PT Concentration % *w*/*w*	Peak Area Ratio Mean ± SD	% RSD
**No variation applied**	0.00833	0.988 ± 0.0042	0.426
	0.00026	0.025 ± 0.0006	2.536
	0.00013	0.014 ± 0.0004	3.274
**Detector temperature (+5)**	0.00833	0.654 ± 0.0316	4.841
	0.00026	0.022 ± 0.0005	2.522
	0.00013	0.0140 ± 0.0005	4.151
**Detector temperature** **(−5)**	0.00833	0.689 ± 0.0189	2.754
	0.00026	0.0208 ± 0.0000	0.108
	0.00013	0.0126 ± 0.0003	2.939
**Oven temperature** **(+5)**	0.00833	0.709 ± 0.0266	3.761
	0.00026	0.0215 ± 0.0002	1.285
	0.00013	0.1341 ± 0.0002	1.827
**Oven temperature** **(−5)**	0.00833	0.7061 ± 0.0183	2.596
	0.00026	0.0235 ± 0.0001	0.554
	0.00013	0.0139 ± 0.0003	2.725
**Flow rate** **(+10)**	0.00833	0.6839 ± 0.0137	2.011
	0.00026	0.0230 ± 0.0003	1.329
	0.00013	0.0134 ± 0.0002	1.827
**Flow rate** **(−10)**	0.00833	0.7720 ± 0.014	1.859
	0.00026	0.0237 ± 0.0003	1.636
	0.00013	0.0139 ± 0.0004	2.908
		Mean	**(2.294)**

**Table 8:** Table with acceptable residual PT data for all injectable hydrogel/filler formulations with different PT concentrations. * Refers to below LOD.

Injectable Hydrogel/Filler Formulation Samples	PT Concentration % *w*/*w* with GC in the Samples	Acceptance of the PT Concentration ˂ 0.008% *w*/*w*
**Freeze-dried Samples**		
**HA-PT 1**	0.000093 * 0.000120 * 0.000123 * Mean 0.00011 SD (± 0.000018)	Accepted
**HA-PT 2**	Not detected	Accepted
**HA-PT 3**	0.000130 0.000141 0.000143 Mean = 0.000140 SD (±0.000007)	Accepted
**HA-PT 4**	0.000146 0.000136 0.000157 Mean 0.000150 SD (±0.000010)	Accepted
**HA-PT 5**	0.023402 0.024990 0.024730 Mean = 0.024370 SD (±0.008000)	Rejected
**Direct Formulation Samples**		
**HA-PT 1**	0.000038 * 0.000040 * 0.000040 * Mean = 0.000040 * SD (±0.000001)	Accepted
**HA-PT 2**	Not detected	Accepted
**HA-PT 3**	0.000060 * 0.000060 * 0.000070 * Mean = 0.000063 * SD (±0.000006)	Accepted
**HA-PT 4**	0.000022 * 0.000030 * 0.000036 * Mean = 0.00003 * SD (±0.000007)	Accepted
**HA-PT 5**	0.024948 0.026236 0.026103 Mean = 0.025760 SD (±0.000700)	Rejected

## Data Availability

Data is contained within the article.
